# A178 BIOLOGIC THERAPY DURING PREGNANCY AS PER GUIDELINE RECOMMENDATIONS REDUCES ADVERSE PREGNANCY RELATED OUTCOMES

**DOI:** 10.1093/jcag/gwab049.177

**Published:** 2022-02-21

**Authors:** J J Tao, V Govardhanam, P Tandon, V Huang

**Affiliations:** 1 University of Toronto, Division of Gastroenterology, Toronto, ON, Canada; 2 University of Toronto, Department of Medicine, Toronto, ON, Canada

## Abstract

**Background:**

Inflammatory bowel disease (IBD) disease activity during pregnancy is associated with adverse neonatal and pregnancy-related outcomes. Biologics are used to suppress disease activity but crosses the placenta in the third trimester. Conflicting studies and guidelines on the timing of biologic dosing in pregnancy persist as we try to balance the risk of disease flare and possible adverse drug effects. The American Gastroenterology Association (AGA) recommends timing the final dose according to the half-life and dosing regimen of each biologic agent.

**Aims:**

To compare neonatal and pregnancy-related outcomes in early versus late dosing of biologics.

**Methods:**

This was a single-center retrospective cohort study conducted at Mount Sinai Hospital from 2016–2021. We included patients with an established diagnosis of IBD before pregnancy who were at least 18 years of age at the time of conception. All patients must have been treated with an IBD-specific biologic agent and had a documented final dose during the pregnancy. The early group received their last biologic dose earlier than the AGA recommendations and the late group received it within the recommended interval. A patient was considered to have a flare based on the overall clinical impression of their gastroenterologist informed by reported symptoms, investigations (fecal calprotectin, endoscopy), and response to treatment. Neonatal and pregnancy-related outcomes were compared amongst the two groups using the student’s t-test (for continuous variables) and Fischer’s exact test (for categorical variables) using SPSS Version 27.

**Results:**

Of 322 patients who had a completed pregnancy at Mount Sinai Hospital, 107 were included in this study. 67 (62.6%) were in the early and 40 (37.4%) were in the late groups. Baseline characteristics including age, comorbidities, IBD phenotype and disease activity were similar between the two groups. The late group had significantly later gestational ages (37.4 vs 38.7 weeks, p=0.006), higher 5-minute Apgar scores (8.7 vs. 9.0, p=0.042), fewer NICU admissions (25.4% vs 5.0%, p=0.036), and fewer IBD flares (28.3% vs 11.1%, p=0.039) in the 6-month post-partum period. There were no significant differences in the rates of premature birth, caesarian sections, infections, and congenital abnormalities. Results are displayed in figure 1.

**Conclusions:**

Our study suggests that late dosing of biologics according to the AGA guidelines was associated with favourable outcomes. However, this is an unadjusted analysis based on retrospective data and findings should be confirmed in a prospective manner to account for confounders.

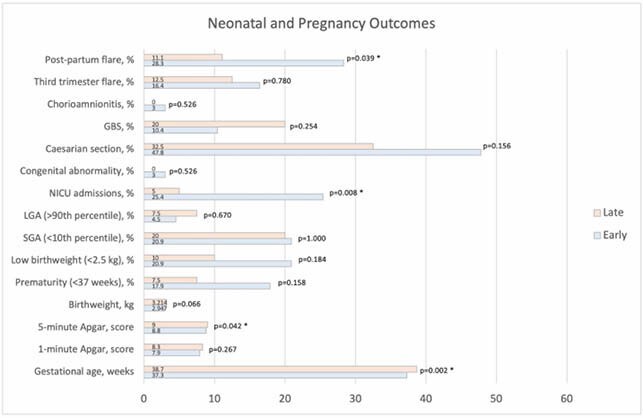

**Funding Agencies:**

None

